# Computer software for a simultaneous multi-element atomic-absorption spectrometer

**DOI:** 10.1155/S1463924682000169

**Published:** 1982

**Authors:** J. M. Harnly, N. J. Miller-lhli, T. C. O'Haver

**Affiliations:** 1 Nutrient Composition Laboratory Beltsville Human Nutrition Research Center Human Nutrition SEA-USDA Beltsville Maryland 20705 USA; 2 University of Maryland College Park Maryland 20742 USA


Journal of Automatic Chemistry, Volume 4, Number 2 (April–June 1982), pages 54–60

### Computer software for a simultaneous multi-element atomic-absorption spectrometer

**J. M. Harnly**

*Nutrient Composition Laboratory, Beltsville Human Nutrition Research Center, Human Nutrition, SEA-USDA, Beltsville, Maryland 20705, USA,*

**N. J. Miller-Ihli and T. C. O'Haver**

*University of Maryland, College Park, Maryland 20742, USA*

### Introduction

A dedicated minicomputer is the essential component of a newly developed simultaneous atomic-absorption spectrometer which uses a continuum source (SIMAAC) [1, 2]. The high-speed data acquisition and analytical calculation capabilities of the computer allow multi-element analyses without the need for an electronic circuit and lock-in amplifier for each channel [3]. The available computing power provides enormous versatility in processing the data. As a result, SIMAAC has analytical characteristics, aside from the multi-element capability, which are unique in the field of atomic-absorption spectroscopy. These characteristics are (1) an extended analytical range for each element covering four to six orders of magnitude in concentration; (2) correction of all computed absorbances for broad-band background absorption; (3) correction for stray light at high analyte concentrations; and (4) application of statistics during the analytical calculations to assess the quality of the results. In addition, the computer prompts the analyst for start-up and run procedures and provides long-term storage of the data and pertinent experimental parameters.

This paper outlines the pertinent parts of the computer porgrams which have been developed for SIMAAC. These programs incorporate the analytical capabilities listed above and make SIMAAC easy to use for routine analyses. A complete listing of the programs is available upon request from the authors.

### Equipment

The physical configuration of SIMAAC, which has been previously reported [1], is shown in figure 1. Briefly, the system consists of a 300W xenon arc lamp (Varian, Eimac Division, San Carlos, California, USA), a Spectraspan III echelle polychromator (Spectrametrics Inc., Andover, Massachusetts, USA) and a dedicated 11/34-VE Declab minicomputer (Digitial Equipment Corporation, Maynard, Massachusetts, USA). Wavelength modulation is performed by a model G-300 PD galvanometer and model CCX-101 Scanner Controller (General Scanning Inc., Watertown, Massachusetts, USA). The minicomputer has 32k words of core memory and is equipped with an analogue-to-digital (A/D) converter (AD11-K); a digital-to-analogue (D/A) converter (AA11-K); a digital input-output (I/O) register (DR11-K); a dual, real-time clock (KWII-K); a floating point processor (FP11-A); a graphics terminal (VT-55-FE); a line printer (LA 180); a fixed, double density (2·5 M word) disc (RKO5F); and a removable cartridge disc (RKO5J, 1·25 M word).

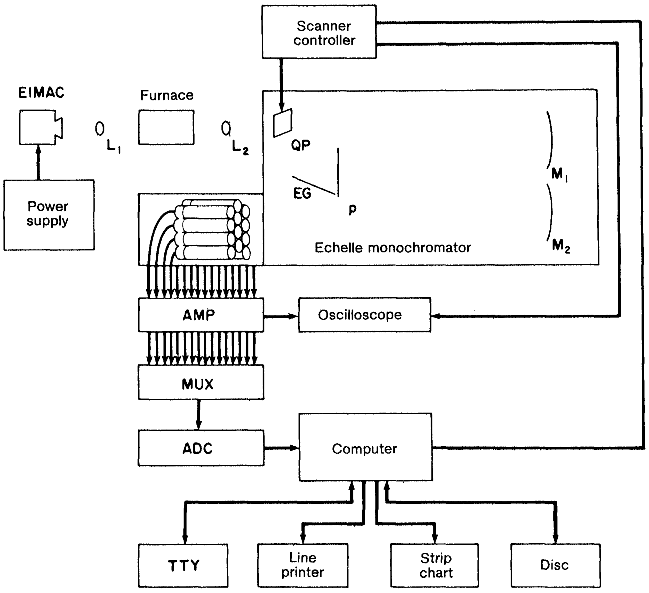

*Figure 1. A block diagram of SIMMAC.*

The 16 photomultiplier tubes (PMTs) of the echelle polychromator are connected to the A/D converter of the computer through the interface amplifier. The interface amplifier is the only SIMAAC component which is not commercially available and consists of 16 parallel operational-amplifier circuits. Each circuit consists of a current-to-voltage converter followed by an inverting amplifer whose variable gain is controlled from the instrument's front panel.

Both flame and electrothermal atomization may be used. Flame atomization employs a burner assembly (Perkin-Elmer Corporation, Norwalk, Colorado, USA) with a 10 cm, single-slot burner (Varian Associates, Palo Alto, California, USA). The electrothermal atomizer is an HGA-2100 graphite furnace and power-supply (Perkin-Elmer Corporation) used in conjunction with an AS-1 auto-sampler. Auto-sampling in the flame atomization mode has also been performed with a flow-injection system described elsewhere [4, 5].

The start of data acquisition is triggered by grounding an input bit on the digital I/O register of the computer. This is accomplished in a number of ways: by using a foot pedal (manual operation, flame atomization); by the furnace power-supply at the start of the atomization cycle (electrothermal atomization); or the autosampler (flow injection, flame atomization). For use with the flow-injection system, the Autosampler

 0142 0453/82/0402 0054 $05·00 © 1982 Taylor & Francis Ltd

II (Technicon Instruments Corporation, Chauncy, New York, USA) was fitted with a solenoid [6] to provide the trigger signal. In the case of electrothermal atomization, the power-supply provides a signal (terminal board 303, pin 1) which goes from 5V to ground at the start of the atomization cycle.

An experiment is terminated by grounding another bit on the digital I/O register. This 'stop' trigger is activated by a toggle switch mounted near the computer.

### Computer programs

Three Fortran programs have been written to accomplish the desired experimental functions. These three programs are: (1) data acquisition; (2) data inspection and editing; and (3) analytical calculations. These programs are accessed one at a time.

### *Data acquisition*

The major functions of the data-acquisition program are shown in the flow chart in figure 2. The data-acquisition program is run for each experiment. An experiment consists of up to 99 runs or atomizations. An atomization consists of acquiring intensity data (step 2) for 1s to 30s (as specified by the analyst before each experiment) while a sample, standard, or blank is atomized in the flame or the graphite furnance. Between atomizations, the intensity data is reduced to absorbance data (step 3) and is stored on the cartridge disc (step 4) to await the analytical calculation program. Steps 2 and 3 are functions normally performed by a computer used for real-time data acquisition for a spectroscopic instrument [7]. In the system described here much higher sampling frequencies are used. Steps 1, 4 and 5 store the experimental data, as well as pertinent experimental parameters for future use. A major system requirement was to make each experimental file complete so that no further information is required to compute analyte concentrations for each element for each sample. After running the data-acquisition program, the analysts can wait as long as desired before calling upon the analytical calculation program to obtain a final sample concentration summary.

The first phase of the experiment start-up routine (step 1) asks the analyst whether flame or electrothermal atomization mode is in use. The software then prompts the analyst to turn on the necessary equipment: the Eimac lamp, the PMT power supply, the interface amplifier, the cartridge disc, the line printer, the scanner controller, the oscilloscope, and the exhaust fans used to vent the atomization source and xenon arc lamp. The program also prompts the operator to turn on either the fuel and oxidant gases (flame atomization) or the cooling water and purge gas (electrothermal atomization). It also reminds the analyst to connect the computer output signal to the scanner controller and to optimize the wavelength setting of the echelle polychromator. The last phase of step 1 is to prompt the analyst to enter the analytical parameters: the modulation amplitude, which elements are 'active' or to be analysed, and the stock-solution concentrations for each element. The requested atomization parameters vary according to the atomization mode. For flame atomization the atomization time, and the pressure, and the flow rate of the fuel and oxidant are requested; while for electrothermal atomization the computer requests the operator to provide the total time, ramp time, and final temperature of the drying, charring, and atomization steps. For electrothermal atomization the program will also request the integration start and stop limits for each element, since the signal is transient. The flame atomization time and the total time of the electrothermal atomization step are the length of time for which the program will acquire data when a start 'trigger' is received. Finally, the

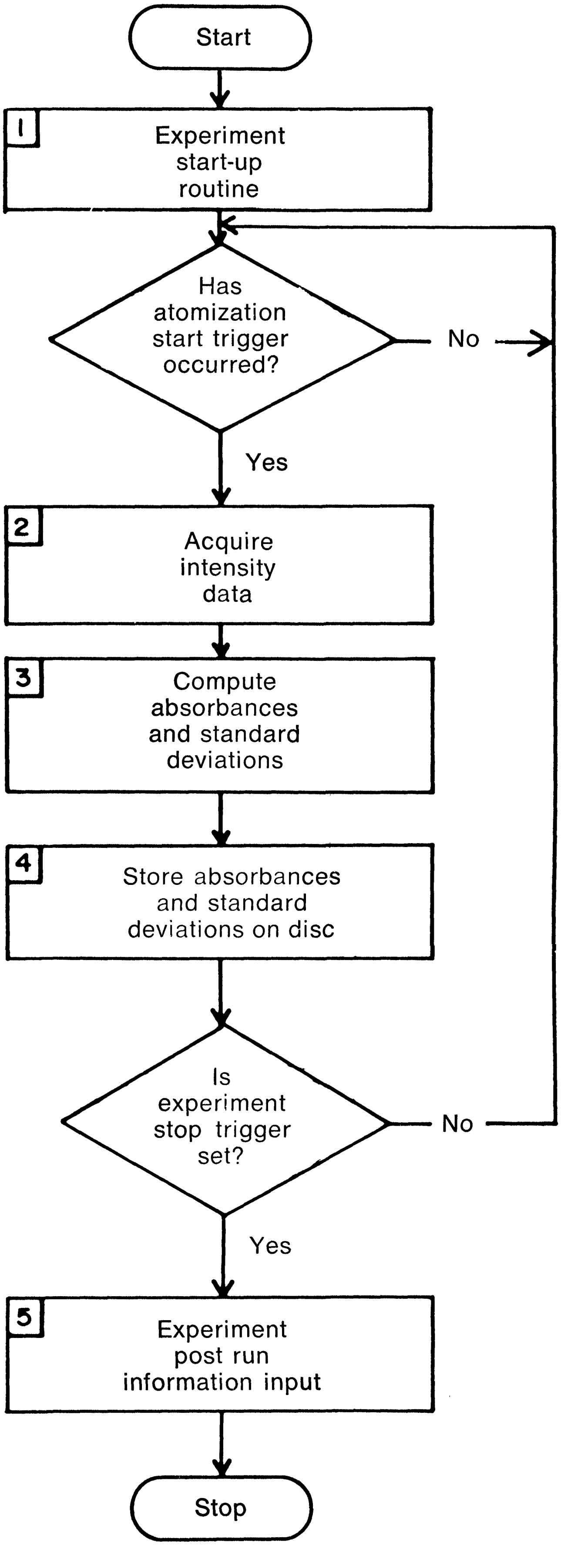

*Figure 2. Data-acquisition flow chart.*

program requires a two-line experiment description to be entered. At this point the program is ready to acquire the intensity data.

The intensity data acquisition and wavelength modulation are accomplished using an assembly language subroutine in response to an 'interrupt' generated by the digital I/O register upon receiving the start 'trigger'. The computer drives the quartz modulator plate (mounted on a galvanometer) located behind the entrance slit [1]. The motion of the modulator plate produces a sweep across each analytical wavelength at a frequency of 56 Hz. During each sweep, or cycle, the computer will acquire 20 intensity data for each element in the following way. The programmable real-time clock is set at a frequency of 1120 Hz (56 × 20). Each clock pulse initiates a sequence of 16 A/D conversions at a rate of 18 kHz. Between each conversion, the program will store the intensity data in a data-storage buffer and will increment the multiplexer's address. Consequently, the computer will cycle through all 16 channels acquiring one word of intensity data for each element. After 16 conversions, the computer will pause and wait for the next clock pulse at which time it will repeat the sequence of 16 conversions. This 'time sliced' data-acquisition scheme is not new [7]. However, the frequency of data acquisition of this program is unusually high for most atomic-spectroscopy techniques; this is essential to obtain the best possible detection limits and to provide the extended analytical range [2].

Immediately following the completion of the intensity data acquisition, the intensity data is reduced to absorbances. Each successive set of 20 intensity data points corresponds to a single pass over the absorption profile of one of the elements. The 20 intensity points are used to compute absorbances in five locations. Each successive absorbance uses intensity data further from the centre of the absorption profile. Consequently, each successive absorbance is less 'sensitive' or has a higher characteristic concentration [2]. A series of standards will produce a family of five calibration curves as shown in figure 3. This family of curves will cover four to six orders of magnitude in concentration, depending on the element. Absorbances are computed only for those elements which the analyst specifies as 'active' at the start of the program.

Absorbances are computed 56 times/s. This frequency minimizes the flicker noise component of the continuum source resulting in a statistical, or 'shot', noise-limited system. SIMAAC detection limits are comparable to those for conventional atomic-absorption spectrometers [2].

For flame atomization, the raw data is ensemble-averaged for 10 passes before the absorbances are computed. That is, 10 sets of 20 intensity data points are averaged point-by-point. For a 5 s atomization time (the standard data acquisition time for flame atomization in this application) 27 sets of five absorbances (at the five locations) are computed. Since flame atomization provides a static signal, the 27 values can be averaged and a standard deviation of the mean computed for the absorbance at each of the five locations. The five averaged absorbances and the five standard deviations of the mean are then stored on the cartridge disc.

When electrothermal atomization is employed, the absorbances at the five locations are computed for each set of 20 intensity data points. The electrothermal atomization signal, unlike the static flame atomization signal, is a pulse. Computing absorbances for each pass across the absorption profile (20 points) produces a response time of 0·018 s (1/56). This is fast

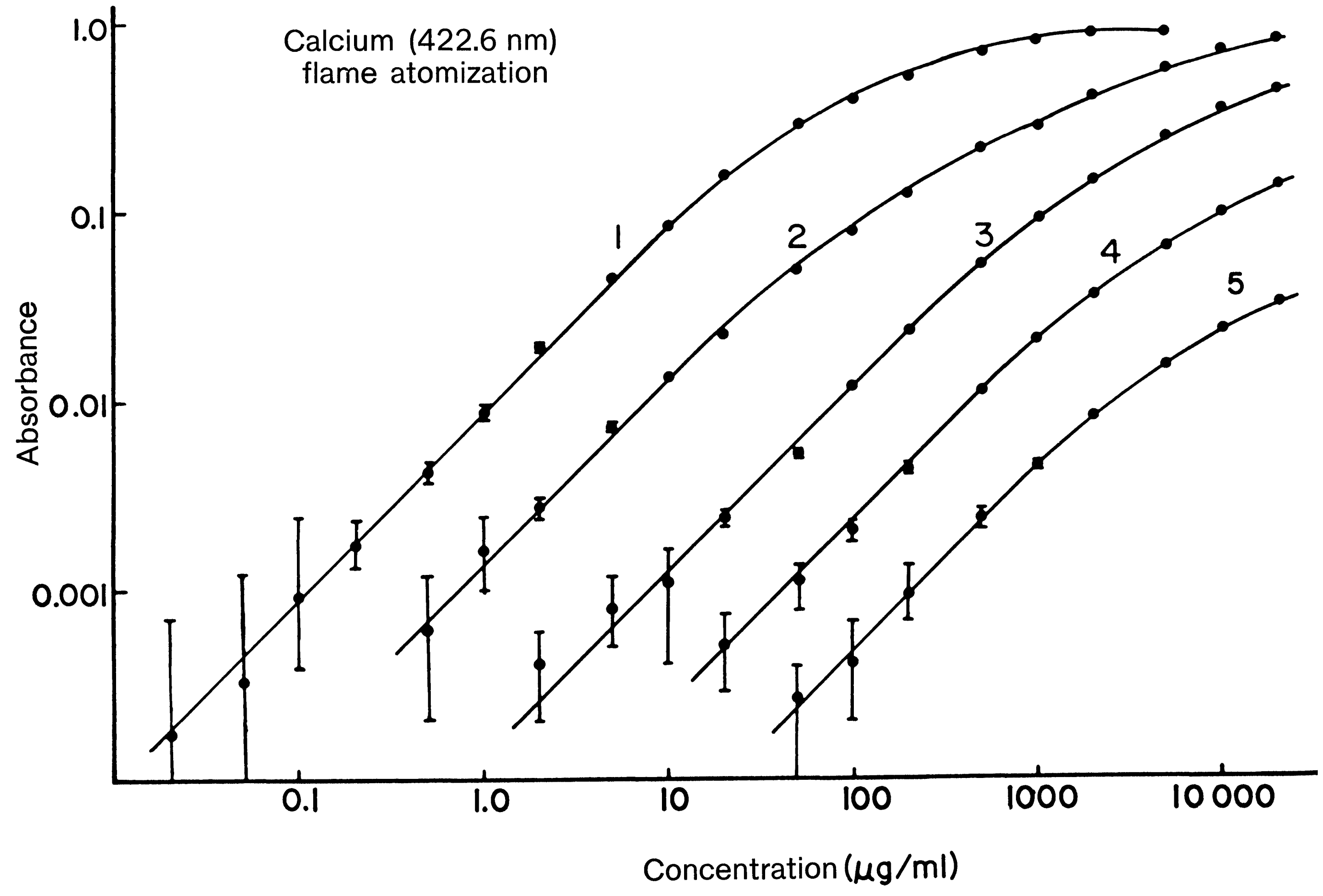

*Figure 3. Family of calibration curves for calcium (442·7 nm) in an air-acetylene flame.*

enough to characterize even the most rapid pulses (due to the most volatile elements) from an electrothermal atomizer. These rapid pulses may have a width at half-height of 0·2 to 0·5 s. For each of the five locations, the maximum or 'peak' absorbance is determined as well as the sum, or integrated absorbance of the pulse. The analyst can specify separate integration intervals for each element at the start of the program. Integration over a short interval around the pulse will optimize the signal-to-noise ratio.

Unlike flame atomization, in the electrothermal atomization technique, a standard deviation is not computed for either the peak area or peak height measurements. The standard deviations for furnace pulse measurements were found to be directly proportional to the analyte signal. For dilute acid standards, a relative standard deviation as low as 1% has been observed. However, this value can become signficantly larger as the sample matrices become more complex.

At the end of the experiment, the analyst activates the stop 'trigger'. The program then prompts the analyst to identify which blank is to be used to compute the base-line standard deviation and to identify each run as a blank, standard, or sample. For each standard, the program also requests a dilution factor (this factor is used with the stock concentrations to compute a concentration for each element for each standard) and a laboratory identification number for each sample.

All experimental parameters and the results of each atomization are stored on the disc cartridge. The program divides the disc into two halves; the first half is used to store the raw intensity data and the second half is used for a file system. The first half is sufficiently large to hold 30 s of data acquisition for 16 elements at a frequency of 1120 Hz. Intensity data from each successive atomization is written over the intensity data of the previous run.

The second half of the disc is organized into a file system which can be randomly accessed. Each cartridge disc has a disc header, which is used to date the cartridge when it is renewed (wiped clean of previous files) and to document the purpose of the set of experiments on that cartridge. Each experiment has an experiment header which is followed by the data from each atomization in that experiment. The experiment header contains the information entered in the experiment start-up routine. Each disc has room for 1306 atomizations.

### Data inspection and editing

The inspection and editing program (figure 4) allows the analyst to (1) list the experiment headers; (2) erase all the data from a cartridge; (3) modify the experimental parameters; or (4) list the absorbance data, by element, for any experiment. It should be stressed that only the experimental parameters can be modified. There is no way the reduced absorbance data can be altered.

Option 1 provides a hard copy print-out of all the experiment headers. The analyst can locate an earlier experiment, check the instrumental parameters used for a previous analysis, or verify that there are no longer any experiments of interest on the disc prior to erasing the cartridge.

If the cartridge is full, and the analyst has finished with the experiments, Option 2 will erase the cartridge, i.e. wipe out all the old files. This is the only way that these files can be eliminated (barring a disc failure). If Option 2 is selected, the program will request the operator to verify the choice. This safeguard prevents the cartridge being erased if the operator accidentally selects Option 2.

Option 3 allows the analyst to modify any experimental parameters which might have been entered by mistake. These parameters include the run identifications (sample, standard, or blank), the stock-solution concentrations, the dilution factors

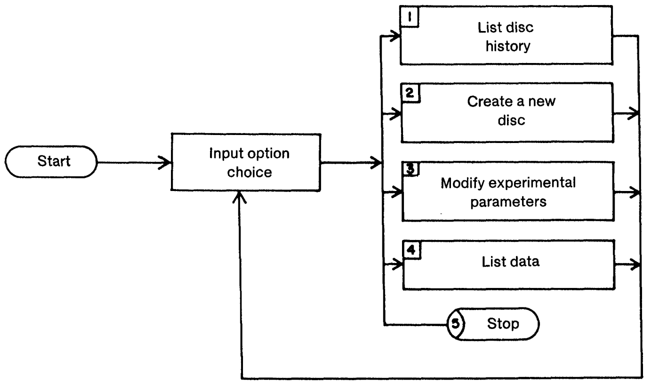

*Figure 4. Data inspection and editing flow chart.*

for each standard, and the blank to be used for computing the base-line standard deviation. The run identifications or labels, besides being changed, can also be erased. If an atomization produces a result which appears to be wrong (for example if the blank is contaminated, if the solution was not aspirated over the entire atomization period, or if an unusually high reading occurs) the analyst can erase the label for every element, or just a single element in the atomization. The data will continue to exist in the file but, since it is no longer labelled, it will not be used in computing the sample concentrations.

Option 4 permits the analyst to inspect all the absorbance data for every atomization and for any experiment on an elemental basis. The data are read-out to the line printer. A detailed inspection of the listings by element is used to detect erroneous results. If the data are satisfactory then the program is halted using option 5 and the analytical calculation program is called.

### Analytical calculations

The data-processing program performs four functions: (1) it averages repeat atomizations; (2) it subtracts the reagent blank; (3) it computes the concentration of the samples from the calibration curves; and (4) it computes the sensitivities and detection limits for each element for the first curve. The various options of this program (figure 5) permit various combinations of these functions to be performed for one or, in sequence, for all of the elements.

Option 1 averages repeat atomizations, subtracts the absorbance of the reagent blank from each standard and sample, and prints this processed data. This is performed for all absorbances at all five locations for all of the 'active' elements. Option 2 performs the functions of Option 1 and then uses the processed data to construct five calibration curves and compute the concentration of all 'active' elements for each sample. A detailed report listing the computed concentration for all five absorbances for each sample and a weighted average of the concentrations is printed. After performing these functions for each element, an experimental summary is printed which reports the concentration of each element for each sample in a tabular format. A second table lists the characteristic concentrations and detection limits for each element computed from the most sensitive curve, curve No. 1.

Option 3 permits the functions of Options 1 and 2 to be performed one step at a time for a single element. A detailed report is printed if the curve fitting and sample concentration computation functions are performed. There is no experimental summary.

Finally, Option 4 permits the analyst to repeat any of the options for a different set of experimental data.

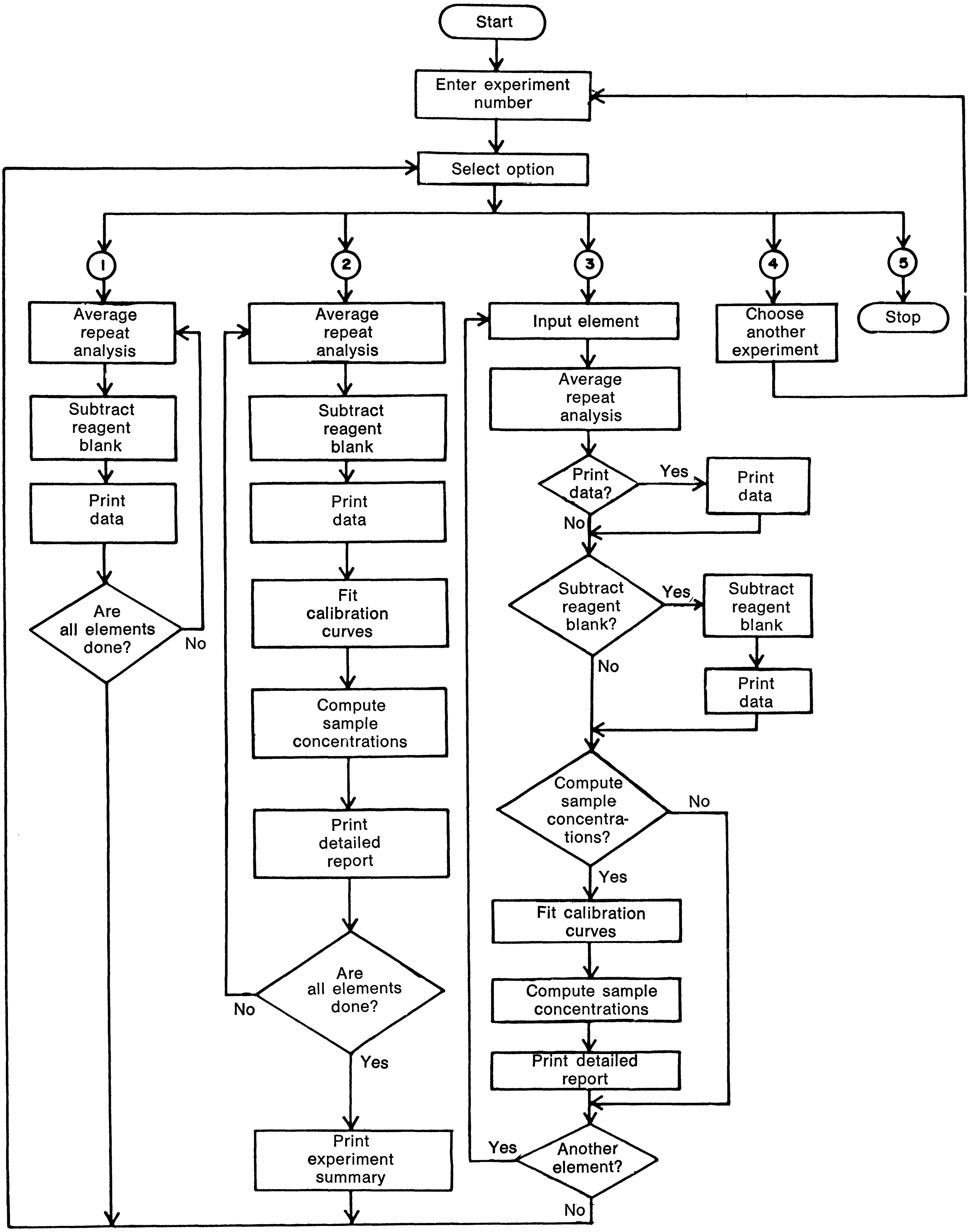

*Figure 5. Data processing flow chart.*

The four main functions of this program require a more detailed examination. The first function, averaging repeat atomizations, is straightforward and requires no further explanation. Then the absorbance of a reagent blank is subtracted from the sample and standard absorbances. This is done for all five absorbances. Separate reagent blanks may be used for the samples and the standards. The dual blank is necessary when the standards are made up in an acid solution and are not taken through the sample digestion procedure. In most cases reagent blank readings are very low, well within the linear range of the calibration curve, making the subtraction of the blank absorbance values valid. If the blank readings are high and fall in the non-linear portion of the calibration curves, the blank subtraction can be performed by computing the concentrations of both the sample and blank and then subtracting the concentration of the blank from that of the sample. This decision must be made by the operator and is not automatically implemented by the program.

The third function of the program is the calculation of sample concentrations from the calibration curves. Since absorbances are computed at each of five locations for each standard, a series of standards will generate a family of five curves [2]. The five absorbances computed for each sample provide the possibility of five concentrations being determined, one from each curve.

The analytical calculation program computes the sample concentrations in several distinct steps. First, the program computes the base-line standard deviation using the designated blank. Second, the program inspects the calibration standards. For each curve for each element the program locates the lowest and the highest 'valid' standards and then makes sure that the absorbances for all standards falling between the two extremes increases monotonically with concentration.

The lowest 'valid' standard is the standard with the lowest concentration whose absorbance exceeds the detection limit by a factor of five. The detection limit is defined as three times the standard deviation of the mean of the base-line absorbance, $\sigma_B$ [8]. At the detection limit, a signal is statistically valid but is not quantitative. The authors decided to establish a threshold equal to five times the detection limit, or $15\,\sigma_B$, which must be exceeded by the absorbance of each standard before it is accepted as quantitative. This corresponds to a relative standard deviation of 6·7% [8].

At the high concentration end of the calibration curves, reversal towards the *X* axis can occur. The highest 'valid' standard is the highest standard for which the absorbance is still increasing. Standards with a higher concentration but lower absorbance are ignored. The standards falling between the highest and lowest valid standards are then inspected to ensure that absorbance increases steadily from the lowest to the highest as a function of concentration. If a steady increase is not observed, if there are not enough data points for the calibration scheme, or if no standards meet these specifications, an error statement is printed and the program continues on to the next calibration curve.

The analytical calibration program performs similar tests on the sample absorbances. If the sample absorbances exceed $15\,\sigma_B$, quantitative results can be reported. If the sample absorbances falls between $3\,\sigma_B$ and $15\,\sigma_B$, a flag is set and the results are labelled 'semi-quantitative'. And if the sample absorbance is less than $3\,\sigma_B$ the sample concentration is reported as less than the computed detection limit. At the upper end of the curve, no concentration is computed if the sample absorbance exceeds that of the highest valid standard. Sample concentrations are computed only for those curves where the sample absorbance falls between the absorbances of the lowest and the highest valid standards. If the sample is sufficiently concentrated such that its absorbance falls on the reverse side of all five calibration curves, no concentrations are computed and a statement is printed stating the sample exceeds the highest valid standard of the least sensitive calibration curve, curve 5.

The program is now ready to fit calibration curves to the standards and to compute sample absorbances. Numerous fitting routines have been used. These include drawing a straight line between the standards which bracket the sample; a least squares fit of a first order (straight line) equation to three or more points; a least squares fit of a quadratic equation (parabolic) to four or more points, with absorbance as a function of concentration and with concentration expressed as a function of absorbance; the rational function described by Limbek *et al.* [9]; and the least squares fit of the quadratic equations and the rational function to the log values of the concentration and of the absorbance data. The best approach is still being evaluated. The versatility of the software and the modularity of this program will allow the best method or methods to be easily implemented. The method used will not influence the present discussion of this program.

A maximum of five concentrations can be determined for each sample, one from each analytical curve. Since there is sure to be some variation between the concentrations, the question arises as to which value is the most accurate or precise. Within the experiment, criterion for precision is the signal-to-noise ratio. The signal-to-noise ratio is most meaningful in terms of concentration since the ratio will reflect not only the ratio of the absorbance signal to the absorbance noise, but the slope of the calibration curve as well. At high absorbances, although the absorbance noise may be relatively small, the calibration curve may be almost horizontal. As a result, a small absorbance error interval may intercept a large concentration interval. An approximation of the signal-to-noise ratio in terms of concentration is achieved by using the calibration curve to convert the sample absorbance and absorbance noise into the corresponding concentration values. In this work each computed sample concentration is weighted by the reciprocal of the concentration variance. The weighted average is then reported as the sample concentration. Semi-quantitative results, results for sample absorbances reading between $3\,\sigma_B$ and $15\,\sigma_B$, are not weighted and are reported only if there are no quantitative results.

The experimental summary presents the weighted concentrations in a tabular format, a sample by element matrix. Four types of results are reported. A number appears with no suffix if a quantitative concentration has been computed. If the number has an asterisk suffix, the number is semi-quantitative. A 'less than' (<) suffix means the sample concentration is less than the detection limit which is printed. A 'plus' (+) suffix means the sample concentration exceeds that of the highest valid standard of the least sensitive calibration curve, curve 5, whose value is printed. A string of asterisks in place of the number means that the sample concentration was not determined.

The experimental summary may be preceeded by a detailed report at the option of the analyst. This report first lists the blank corrected absorbances of the standards, in order of concentration, and of the samples. The report then proceeds to list the computed concentration of each sample from each curve with the weighting factors and the final weighted average concentration. This can be obtained for just one element or for every element depending on which option has been selected.

Finally, the fourth major function of the program is to compute the sensitivity (characteristic concentration) and detection limit for each of the 'active' elements from the calibration standards for curve 1, the most sensitive curve. This data is listed in a second table following the experimental summary. Since the calibration standards are used to compute the sensitivity and detection limit, these values will not reflect the effect of the sample matrix on any element. However, these data permit the operator to determine whether optimum instrument parameters were used and to compare instrument performance between experiments.

### Execution times

The execution time of the data-acquisition program has been closely examined in order to minimize the time between atomizations. A comparison of the flame and electrothermal data-acquisition programs for typical operating conditions is shown in table 1. Flame atomization usually employs a 5 s integration. Since all 16 elements are sampled 1120 times/s, a total of 89 600 intensity data points are acquired in 5 s. With electrothermal atomization, a ramped atomization cycle is used which takes 10 s to reach the maximum temperature (2800°C according to the front panel meter) and then rests at this temperature for 3 s. A total of 232 960 intensity data points are acquired during the 13 s atomization period.

*Table 1. Execution times for a typical atomization.*

| | Flame atomization | Electrothermal atomization |
|---|---|---|
| (1) Data acquisition | 5·0 s | 13·0 s |
| (2) Raw data read | 6·6 s | 17·2 s |
| (3) Data reduction | | |
| (*a*) 1 element | 0·8 s | 5·0 s |
| (*b*) 16 elements | 13·6 s | 80·0 s |
| Subtotal (16 elements) | 25·2 s | 110·2 s |
| (4) Print | | 19·0 s |
| Total (16 elements) | 25·2 s | 129·2 s |

The reduction of the intensity data to the 10 data values per element which are stored in the file system is dependent on several factors as shown in table 1. First, it takes time to read the intensity data from the cartridge disc back into core. The time required corresponds to 74 ms for each 1000 intensity data points.

Secondly, the intensity data must be reduced to absorbance data and either averaged or integrated. In the case of flame atomization, the intensity data from 10 passes across the absorption profile are ensemble averaged before the five absorbances are computed. After 270 passes, the average and standard deviation for the absorbance at each of the five locations is computed. The reduction time is 0·17 s per element/s of atomization. Electrothermal atomization requires 0·38 s per element/s of atomization. In this time absorbances at each of the five locations are computed for every 20 intensity points. The data are integrated over the desired interval and the maximum absorbance is determined for each of the five absorbances. For both atomization modes, the data reduction time is directly proportional to both the length of the atomization and to the number of elements analysed.

Lastly, the data must be displayed. For electrothermal atomization the five peak areas and five peak heights are printed by the line printer. This step takes 19 s, resulting in a total delay of 116 s between atomizations (129 – 13) when 16 elements are analysed. However, the data reduction can take place concurrently with the drying and charring steps of the next atomization. Lengthy drying and charring steps are necessary to ensure gentle evaporation of the sample and elimination of as much of the sample matrix as possible. Electrothermal atomization seldom occurs at a frequency greater than one atomization every 2 min. for conventional, single-element analysis. Consequently, the additional delay (data reduction time – drying and charring time) imposed by the data reduction is not usually significant.

A delay is more noticeable for flame atomization. To reduce the execution time, the data are not printed for each atomization. Instead, the most sensitive absorbance (curve 1) for the element designated to be monitored is displayed on the video terminal. This means that after a standard 5 s atomization, the program requires a delay of 20 s before the next atomization can take place. This delay has been found not to be limiting if samples are aspirated manually. Generally the computer is waiting for the analyst who must recap the sample or standard, note the identity of the solution just atomized, and uncap the next solution to be atomized. As a result, the theoretical rate of one atomization every 25 s can only be achieved if automatic sample injection is used.

The time required for the analytical calculations (averaging repeat analyses, blank subtraction, curve fitting and computing sample concentrations) at the end of an experiment will vary drastically depending on whether or not a detailed report is obtained. A summary table can be obtained for 30 samples analysed for 12 elements in less than 5 min. A detailed report for the same experiment requires 30 min.

Experience in recent months, indicates that an experiment involving the analysis of 30 samples, by flame atomization, from the start of atomization to the generation of the experimental summary, requires 60 min. or less. Each experiment involved a single atomization of all 30 samples, duplicate atomizations of the eight standards, and the atomization of all the necessary water and reagent blanks. Twelve elements were determined using 5 s atomizations. A detailed report was not requested; only the experimental summary was printed. The highest throughput on a single day has been three such experiments resulting in 1080 determinations (3 experiments × 30 samples × 12 elements). These experiments were performed without automatic sample injection. Consequently, the current limiting factor is the endurance of the analyst, not the execution time of the various programs.

### Acknowledgement

Part of this paper was presented at the 7th Annual Meeting of the Federation of Analytical Chemistry and Spectroscopy Societies, Philadelphia, Pennsylvania (1980). The material is taken from a dissertation to be submitted to the Graduate School, University of Maryland by N. J. Miller-Ihli, in partial fulfilment of the requirements for the Ph.D. degree in chemistry.

Mention of trademark or proprietary products does not constitute a guarantee or warranty of the product by the US Department of Agriculture and does not imply their approval to the exclusion of other products that may also be suitable.

### References

1. Harnly, J. M., O'Haver, T. C., Golden, B. and Wolf, W. R., *Analytical Chemistry*, **51** (1979), 2007.
2. Harnly, J. M. and O'Haver, T. C., *Analytical Chemistry*, **53** (1981), 1291.
3. Harnly, J. M. and O'Haver, T. C., *Analytical Chemistry*, **49** (1977), 2187.
4. Wolf, W. R. and Stewart, K. K., *Analytical Chemistry*, **51** (1979), 1201.
5. Wolf, W. R. and Harnly, J. M., Paper No. 347, Pittsburgh Conference on Analytical Chemistry and Applied Spectroscopy, Atlantic City (10–14 March 1980).
6. Stewart, K. K. and Rosenfeld, A. G., *Journal of Automatic Chemistry*, **3** (1981), 30.
7. Haas, W. J., Jr., Fassel, V. A., Grabau, F. IV, Kniseley, R. N. and Sutherland, W. L. Simultaneous determination of trace elements in urine by inductively coupled plasma-atomic emission spectrometry. In *Ultratrace Metal Analysis in Biological Sciences and Environment*, T. H. Risby (ed.) No. 172, Advances in Chemistry Series (American Chemical Society, 1979).
8. Currie, L. A. *Analytical Chemistry*, **40** (1968), 586.
9. Limbek, B. E., Rowe, C. J., Wilkinson, J. and Routh, M. W., *American Laboratory*, **10** (1978), 89.

